# Isolation and characterization of porcine epidemic diarrhea virus associated with the 2014 disease outbreak in Mexico: case report

**DOI:** 10.1186/s12917-016-0763-z

**Published:** 2016-06-29

**Authors:** María Elena Trujillo-Ortega, Rolando Beltrán-Figueroa, Montserrat Elemi García-Hernández, Mireya Juárez-Ramírez, Alicia Sotomayor-González, Erika N. Hernández-Villegas, José F. Becerra-Hernández, Rosa Elena Sarmiento-Silva

**Affiliations:** Departamento de Medicina y Zootecnia de Cerdos, Facultad de Medicina Veterinaria y Zootecnia, Universidad Nacional Autónoma de México, Mexico City, 04510 Mexico; Departamento de Microbiología e Inmunología, Facultad de Medicina Veterinaria y Zootecnia, Universidad Nacional Autónoma de México, Mexico City, 04510 Mexico; Departamento de Patología, Facultad de Medicina Veterinaria y Zootecnia, Universidad Nacional Autónoma de México, Mexico City, 04510 Mexico

**Keywords:** Porcine epidemic diarrhea virus, Mexico, Outbreak, Characterization

## Abstract

**Background:**

Interest in porcine epidemic diarrhea has grown since the 2013 outbreak in the United States caused major losses, with mortality rates up to 100 % in suckling piglets. In Mexico, an outbreak of porcine epidemic diarrhea, characterized by 100 % mortality in piglets, began in March 2014 in the State of Mexico.

**Methods:**

The aim of this study was to confirm and identify porcine epidemic diarrhea virus (PEDV) in samples from piglets with suggestive clinical signs using virological, histological, and molecular techniques. Necropsy was performed on 13 piglets from two litters with initial and advanced clinical signs. Suggestive lesions of acute infection with PEDV were detected in histological sections of the small and large bowels; specifically, multiple virus particles with visible crown-shaped projections were observed using electron microscopy and negative staining. Viral isolation was performed in Vero cells with trypsin. Infection was monitored by observation of cytopathic effect, and titration was determined by TCID_50_/ml. The presence of the PEDV in cultures and clinical samples was confirmed by RT-PCR amplification and sequencing of a 651-bp segment of the S glycoprotein gene, as well as a 681-bp matrix protein gene.

**Results:**

The nucleotide sequence analysis of the Mexican isolates showed marked homology to viruses that circulated in 2013 in Colorado, USA.

**Conclusions:**

In this paper we confirm the isolation and characterization of PEDV from animals with early and advanced clinical signs.

## Background

Recently, there has been a growing interest in porcine epidemic diarrhea (PED), stemming from the major losses caused by the 2013 outbreak in the United States, with mortality rates up to 100 % in suckling piglets [1–5]. The infectious agent causing the outbreak was identified on May 10, 2013, at the Veterinary Diagnostic Laboratory of Iowa State University in Ames. Infection by a coronavirus-like virus, known as porcine epidemic diarrhea virus (PEDV), was confirmed [6, 7].

The disease first obtained recognition as a devastating enteric disorder affecting pigs in the UK in 1971. However, it wasn’t until 1978, in Belgium, that the etiologic agent was identified as a coronavirus and given the name PEDV (PEDV strain CV777). While the disease was first reported in the United Kingdom in 1970, it has since then spread to Belgium, Hungary, Korea, Italy, Thailand, Japan, and China. In Asia, PED has been considered endemic since 1982, causing substantial economic losses to the swine industry [6, 8–10].

PEDV is an Alphacoronavirus classified in the subfamily *Coronavirinae* of the family *Coronaviridae*. PEDV is an enveloped, single-stranded, positive sense RNA virus that infects swine, usually causing respiratory and gastrointestinal disease [2, 8, 9, 11]. The complete genomic sequence of PEDV has a length of 28,038 nucleotides (nt) starting with a 292 nt untranslated region (UTR), followed by six genes—replicase (Rep), spike (S), ORF3, envelope (E), membrane (M), and nucleoprotein (N)— and ending with a 3'untranslated region from 27 706 to 28 038 nt [9].

PEDV infects the epithelium of the small intestine, an environment rich in proteases, and causes atrophy of the villi resulting in diarrhea and dehydration. Therefore, this disease is characterized, as its name implies, by diarrhea, often watery, as well as some systemic signs such as vomiting, fever, anorexia, and lethargy. The disease is more severe in suckling piglets because of their increased susceptibility to dehydration, but outbreaks are also observed in growing pigs and occasionally in adults [12].

In terms of diagnosis of PEDV infection, there are reports of veterinary diagnostic laboratories that have developed molecular detection techniques. However, viral isolation in cell culture remains the confirmatory test. This procedure is considered difficult to perform due to specific conditions required by the virus, such as trypsin supplementation [6, 13].

Although there have been studies describing the disease in the United States and Canada, to our knowledge, this is the first report of PEDV in Mexico [1, 10, 14]. The aim of this study was to isolate, identify, and characterize PEDV from samples collected during an outbreak that occurred in Mexico in 2014.

## Methods

The outbreak began on March 22, 2014, and samples were taken two days after. Directed sampling was performed in pigs in the weaning phase experiencing diarrhea, vomiting, and dehydration that caused 100 % mortality in piglets with permission of the owner. On April 2, samples of lung, gastric contents, stomach, intestine (duodenum), and intestinal contents (feces) were taken from 5 piglets in a litter with early clinical signs (litter 1, ID C1, 6 h of age), and five piglets in a litter with advanced clinical signs (litter 2, ID C2, 24–36 h of age). Euthanasia of both litters was performed by electrocution and subsequent exsanguination (AVMA Guidelines for the Euthanasia of Animals: 2013 Edition). In addition, two samples (lung, gastric contents, stomach, and duodenum) from dead piglets (RIP1 and RIP2) that were 36–48 h old at the time of death, as well as feces from two finishing pigs with diarrhea, were taken.

### Histopathology

Samples of lung, stomach, small intestine (duodenum, jejunum and ileum), large intestine (cecum and colon), and mesenteric lymph nodes were collected and preserved in 10 % formalin buffered at pH 7.2. These samples were subsequently processed by routine paraffin embedding technique and staining with hematoxylin and eosin [15]. Tissues were evaluated using Leica DM500 optical microscopy.

### Transmission electron microscopy

Fragments of small intestine (jejunum) were fixed in 2.5 % glutaraldehyde for 24 h, washed with a cacodylate solution buffered to pH 7.2. Then were treated with 1 % osmium tetroxide, and washed with collected cacodylate buffer. Subsequently, they were dehydrated with increasing concentrations of acetone and embedded in epoxy resin. Semi-thin sections (200 nm) were cut and mounted on slides and contrasted with toluidine blue. Finally, fine cuts of 60 nm were mounted on copper grids and contrasted with uranyl acetate and lead citrate for posterior observation and evaluation in a Zeiss EM 900 electron microscope.

### Viral isolation

Pools of samples including stool and bowel from litter 1 (advanced clinical signs), and litter 2 (initial clinical signs) were macerated by adding 5 ml of D-MEM (Gibco Cat. 10313-021 Lot. 1374740) in the presence of antibiotics (Penicillin 10.00 U/ml, streptomycin 10,000 mcg/ml Gibco Cat. 15140-122 and 200 mM L-glutamine, Gibco 25030-081), and centrifuged at 1500 rpm for 10 min. The supernatant was filtered using a Millex GP filter unit, 0.22 μm (Millipore Express PES membrane, Cat. SLGP033RB).

Vero cells were grown in six-well plates (Corning Inc. COSTAR 3527) previously washed with D-MEM (5 times) to remove the fetal bovine serum. These plates were then inoculated with filtered supernatants in D-MEM supplemented with different concentrations of trypsin (2.5, 5, 10, and 20 μg/ml, and 2 mg/ml) (DifcoTripsine 250 Cat. 215240, Lot. 4181462). After 2 h, this material was removed and the plates were filled with fresh complete medium containing trypsin.

At 48 h post-infection the cells were resuspended, collected, sonicated for 5 min at 37 °C (BransonicSonifier, 5510 Ultrasonic cleaner), and centrifuged at 5000 rpm. An aliquot of 500 μl was collected for posterior RNA extraction. The remaining supernatant was used to infect serial passages in Vero cells in the above conditions (Passage 2).

Supernatant titrations were performed with TCID_50_/ml, using Reed & Münch formula [16].

### RT-PCR

#### RNA extraction

RNA extraction from different passages of the supernatants and samples (intestine, feces, gastric contents, stomach) was achieved with Trizol reagent according to the manufacturer’s instructions (Gibco Life Technologies Cat. 15596-018). Table 1 shows the conditions used in the one step RT-PCR reaction.

**Table 1:** Conditions used for the RT-PCR

ID	Gene	Sequence	Position	Tm °C	Amplicon size
PEDVF (1)	S^a^	TTCTGAGTCACGAACAGCCA	1,466	55	651
PEDVR (1)	S^a^	CATATGCAGCCTGCTCTGAA	2,097	55	651
MPED2F (2)	M^b^	AGTCTTACATGCGAATTGACC	2,565	55	681
MPED2R (2)	M^b^	AGCTGACAGAAGCCATAAAGT	2,398	55	681

^a^[20], ^b^[21]

#### RT-PCR conditions

RT-PCR reaction was performed using Onestep Kit (Qiagen Cat. 210212) with some variations for a total 20 μl reaction mixture: 4 μl 5× Buffer, dNTPs 0.8 μl, 0.8 μl Primer, 0.8 μl Primer R, 0.8 μl enzyme mixture, 0.4 μl RNase inhibitor, 7.4 l of H_2_O, and 5.0 μl of RNA.

The thermocycler was preheated to 50 °C for 2 min, after which the reaction tubes were placed, reverse transcription was carried out at 50 °C for 30 min, and reverse transcriptase was inactivated for 15 min at 95 °C. The reaction was carried out under the following conditions: 94 °C 2 min, (94 °C 1 min, 55 °C 1 min, 72 °C 90 min) for 40 cycles, with a final period of 10 min at 72 °C. Selected primers and their specifications are shown in Table 1.

In order to confirm the presence of viral antigens and to discard the possibility of TGEV and rotavirus infection, Immunochromatography in Sandwich PEDV Ag (Bionote PED Ag Cat. RG14-01), TGE Ag (Bionote TEG/PED Ag Cat. RG14-03), and Rotavirus Ag test (Bionote PED/ROTA Ag Cat. RG14-05) were performed according to manufacturer instructions in gastric content, feces, lung, and intestine samples of one litter as well as in cell culture supernatant of VERO infected cells.

#### Sequencing

The amplification products were purified by SE-Gel® 2 % (Size Select Agarose Gels™, Invitrogene Cat. G6610-02) according to the manufacturer’s protocol and sequenced by the Sanger method using ABI 3130 platform Sequencing. The sequences were edited, assembled, and aligned using Bio-Edit, Clone Manager Version 9, MultAlin 5.4.1, and MEGA6 programs.

## Results

### Gross lesions

**Fig. 1:**
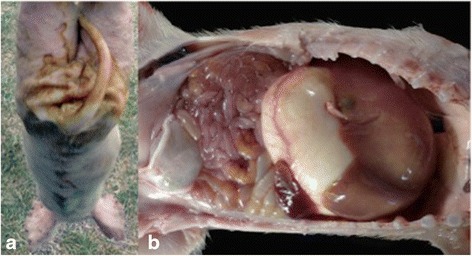
**a** Suckling pig, 6 days old. The perianal region exhibits abundant yellow watery stools. **b** Abdominal cavity. The stomach is markedly dilated and full of coagulated milk. Small bowel dilatation and thinning, through which abundant yellow water content and undigested food remains can be observed

Necropsy was performed on piglets less than one week old from two litters, all of which exhibited a body condition of 1–3/5, anorexia, depression, dehydration, and watery yellow diarrhea. Postmortem examination in 20 % of the piglets showed small areas of lung consolidation in the cranial lobes. The stomach was markedly dilated and full of coagulated milk. 100 % of piglets exhibited small bowel dilatation and in the intestinal lumen, abundant water content and yellow coagulated milk was present (Fig. 1).

### Microscopic lesions

**Fig. 2:**
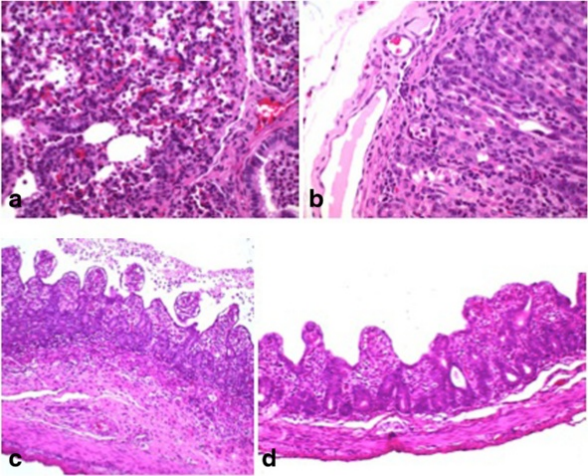
**a** Lung. Slight lymphohistiocytic interstitial infiltrate is observed, as well as moderate neutrophilic infiltrate intra-alveolar bronchiolar. **b** Stomach. The gastric mucosa parietal cells exhibited necrosis and multifocal neutrophilic infiltrate with mild dilatation of lymphatic vessels. **c** and **d** Jejunum. Degeneration and necrosis of intestinal epithelial cells, severe villous atrophy, and mild to moderate lymphocytic infiltrate can be observed

Histological sections of lung, stomach, small intestine (duodenum, jejunum and ileum), large intestine (cecum and colon), and mesenteric lymph nodes were evaluated. In the lung, mild interstitial lymphohistiocytic infiltrate was observed; as well as neutrophilic infiltrate intra-alveolar bronchiolar moderate. In the gastric mucosa, parietal cell necrosis, neutrophilic and lymphocytic infiltrate, and mild multifocal and dilated lymphatic vessels were seen. In histological sections of small and large intestine, degeneration and necrosis of intestinal epithelial cells, severe villous atrophy, lymphocytic infiltrate, mild to moderate congestion, and dilatation of lymphatic vessels were detected. The most significant changes were noted in the jejunum and ileum. Finally, the mesenteric lymph nodes exhibited moderate lymphoid hyperplasia (Fig. 2).

### Transmission electron microscopy

**Fig. 3:**
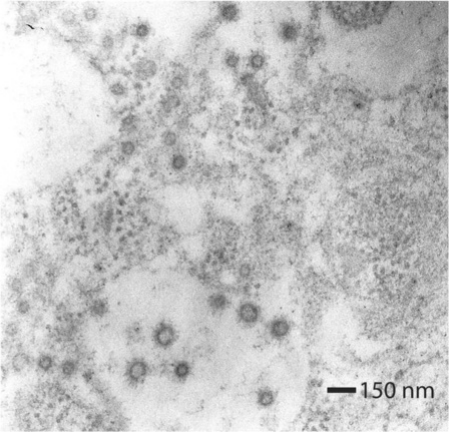
Electronic transmission photography of an enterocyte’s cytoplasm. Numerous viral particles measuring 75–83 nm in diameter are observed. These viral particles possess a membrane with numerous slightly electrodense projections that are 20 nm in length (*arrow*). Adjacent to these viral particles, clusters of ribosomes (*inset*) are appreciated. Contrast technique with uranyl acetate and lead citrate. 50,000× magnification

Ultrastructural evaluation of the small intestine of piglets from both litters was accomplished. Enterocytes exhibited shortening and degeneration of microvilli. In the cytoplasm, the rough endoplasmic reticulum exhibited different degrees of expansion and loosening of ribosomes, and mitochondrial cristae were lost due to swelling. Numerous spherical viral particles were observed, with a size and morphology compatible with the described characteristics of coronavirus structure (Fig. 3).

### Viral isolation and characterization

In the first passage, Vero infected cells with 2 mg/ml and 20 μg/ml of trypsin were detached, as well as uninfected cells with the same concentrations of trypsin. Infected cells treated with 2.5, 5, and 10 μg of trypsin showed cytopathic effect (CPE), which consists of rounded cells, small plaques, intracytoplasmic vacuoles, and detachment not observed in uninfected cells. The effect was observed 24 h post-infection in cells infected with samples from litter 2.

**Fig. 4:**
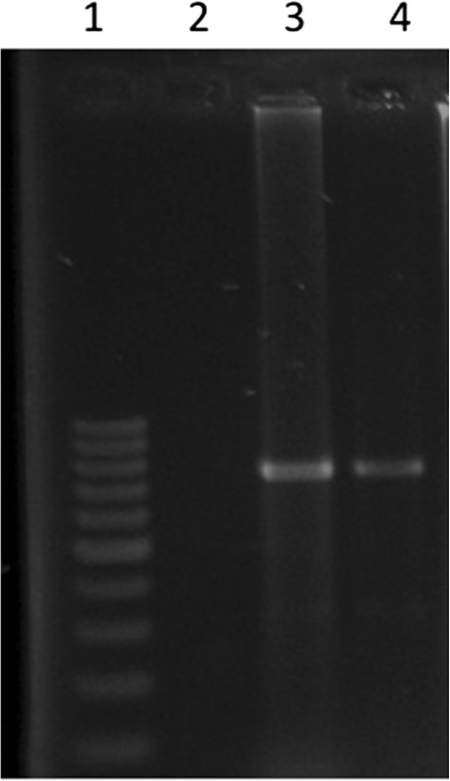
RT-PCR PEDV M gene. *1*. Molecular size 100-bp, *2*. Vero cells (NC), *3*. RT-PCR of M gene from a gastric content sample and *4*. RT-PCR of M gene from supernatant of passage 9 of infected Vero cells

For subsequent passages, trypsin concentrated at 10 μg/ml was selected, as the cells did not detach, CPE was observed, and identification by RT-PCR was positive (Fig. 4).

**Fig. 5:**
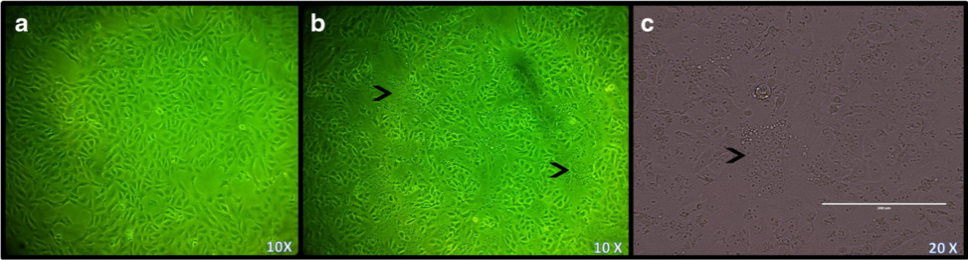
CPE representative effect from passage 9 after 24 h post-infection. **a** Negative control 10×. **b** Passage 9 from Vero cells infected with gastric content 10×. and **c** Passage 9 from Vero cells infected with gastric content 20×

A total of three passages of virus isolates in Vero cells were carried out to determine whether the isolated virus could propagate and survive efficiently in cell culture. A clear CPE was observed after 24 h post-infection. Figure 5 shows a representative effect from passage nine after 24 h post-infection. During these passages the title of infectious virus fluctuated between 2.32E +03 and 2.81E + 06 TCID_50_/ml.

### Molecular diagnosis

#### RT-PCR in clinical samples

The S gene fragment of 651-bp was amplified with PEDVF and PEDVR primers, (Table 1) while the primers MPED2F and MPED2R amplified a M gene fragment of 681-bp. Table 2 summarizes RT-PCR results in different types of samples, as well as direct Immunochromatography in Sandwich results of PED, TGE, and Rotavirus Ag tests, confirming the presence of PEDV and the absence of TGEV and porcine rotavirus antigens.

**Table 2:** Summary of RT-PCR results for the amplification of fragments M and S from different tissues and Immunochromatography in Sandwich PEDV, TGEV, and porcine rotavirus antigen (Ag)

SAMPLE	RT-PCR M			RT-PCR S			ISOLATE			Ag test		
										Rotavirus	PEDV	TGEV
	C1	C2	RIP	C1	C2	RIP	C1	C2	RIP			
STOMACH CONTENTS	+	+	+	+	+	+				-	+	-
INTESTINE	+	+	+	+	+	+	+	+	+	-	+	-
STOMACH	+	+	+	+	+	+				-		-
FECES	+	+	N.C.	+	+	N.C.	+	+		-	+	-
LUNG	-	-	-	-	-	-				-	-	-

*NC* not collected

#### Nucleotide sequence accession numbers

The 4 partial sequences of each gene (M and S) were deposited in GenBank under accession numbers KM044328, KM044329, KM044330, KM044331, KM044332, KM044333, KM044334, and KM044335, respectively.

**Fig. 6:**
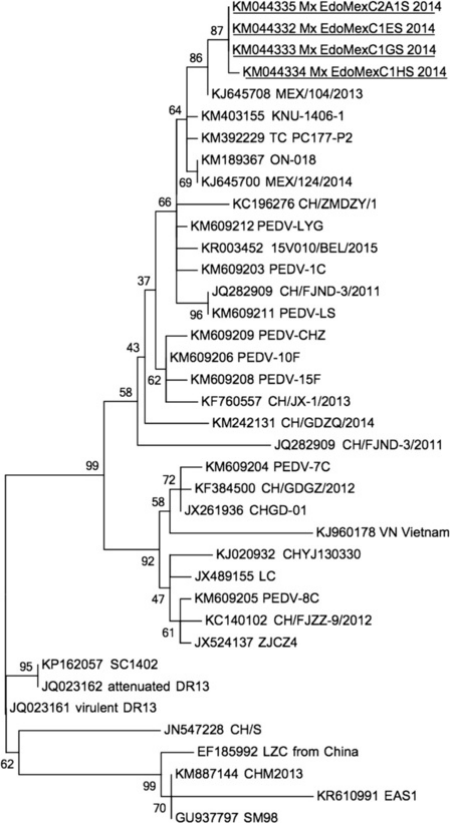
Phylogenetic tree based on Spike gene fragment obtained. Constructed by using the maximum-likelihood method in the MEGA6 program. 1000 Bootstrap

Molecular analysis was completed with partial amplifications of S and M viral genes; the sequences obtained for S gene (593-bp) showed 99 % similarity with the same region of the strains NPL-PEDV/2013/P10 (KJ778616, Lawrence PK unpublished date May 2014), K13JA11-4 (KJ539153, Cho, YY. 2014), and OH14 (access No. KJ408801). For M gene (681-bp) 95 % similarity was found with strains NPL-PEDV/2013/P10 (KJ778616 [17], OH14 (access No. KJ408801), and USA/Indiana/17846/2013 (Access No. KF452323) (Fig. 6) [18].

## Discussion

Enteric diseases in piglets cause severe losses in the swine industry. Infectious agents commonly involved are *Escherichia coli, Clostridium perfringens* type C, *Isospora suis*, rotavirus, and coronavirus (TGEV and PEDV). Recently, the porcine epidemic diarrhea virus (PEDV) has become highly relevant as a result of the outbreak detected in the United States in 2013 [19].

In Mexico, the National Health, Food Safety, and Food Quality Service (SENASICA), sent a notification in May 2014 to the OIE alerting the existence of suggestive clinical evidence indicating the presence of PEDV. Official veterinary services, in coordination with farmers, developed a diagnostic protocol for the disease, including epidemiological sampling of finishing, fattening, breeding stock, and backyard farm animals exhibiting clinical signs. In the research conducted from August 2013 to May 2014, 2309 samples were analyzed by Real Time PCR, of which 30 % were positive. It should be noted that, until that report, authorities had not been able to isolate PEDV, so participation of other infectious agents cannot be ruled out.

In the present study, the clinical symptoms, morbidity, mortality, as well as macroscopic and microscopic lesions observed, coincided with field and experimental infections with PEDV [2, 5, 7, 8, 12]. Moreover, we detected the presence of viral particles compatible with coronavirus in jejunal enterocytes using transmission electron microscopy. These findings, along with the absence of histopathological evidence of bacterial and parasitic pathogens such as *Escherichia coli, Clostridium perfringens* type C, and *Isospora suis,* allowed us to discard possible differential diagnoses to TGEV and porcine rotavirus, which were ruled out using the antigen detection test.

This is the first study reporting isolation of PEDV from the outbreak in Mexico. Isolation from intestine and fecal samples of two litters of animals with initial and advanced clinical signs was achieved using several concentrations of trypsin: 2.5, 5, 10, 20 μg/ml, and 2 mg/ml. Uninfected cells treated with 20 μg/ml and 2 mg/ml detached from all culture bottles; 5 and 10 μg/ml treated cells remained attached allowing observation of cytopathic effect after 24 h post-infection. These results show that using a lower quantity of trypsin (15 μg/ml) than reported in other studies still allows the virus to infect the target cells [13].

RT-PCR was performed to confirm the presence of the virus in cell culture with the amplification of viral genes S and M as reported by Kim et al. (2001) and Li et al. (2012), with observation of expected amplification products throughout all passages [20, 21]. Likewise, titration of each passage was obtained via TCID_50_/ml. Titers ranging between 2.32x10^3^ and 2.81x10^6^ TCID_50_/ml demonstrate progressive replication throughout serial passages.

PEDV isolation represents an important tool for the investigation of its pathogenesis, as well as for the development of serological and molecular diagnostic techniques. A standardized technique for PEDV propagation is essential for potential development of an attenuated vaccine that could be used in nursery pigs and pregnant sows to mitigate the negative impact caused by the disease.

## Conclusions

The pigs described in this study already showed clinical signs consistent with the PEDV infection as we demonstrated by differential diagnosis and in this paper we confirm the isolation and characterization of PEDV from animals with early and advanced clinical signs.

## Abbreviations

AVMA, American Veterinary Medical Association; CPE, cytopathic effect; OIE, World Organization for Animal Health; PCR, polymerase chain reaction; PED, porcine epidemic diarrhea; RT, reverse transcriptase; TGE, transmissible gastroenteritis
